# Supplementary material for: Trans-Regulation of Alternative PD-L1 mRNA Processing by CDK12 in Non-Small-Cell Lung Cancer Cells
Source: Cells. 2023 Dec 15;12(24):2844. doi: 10.3390/cells12242844 (PMC10741404; doi:10.3390/cells12242844)
Supplement: Supplementary file 1 [file cells-12-02844-s001.zip › cells-2695102-supplementary.pdf]

Supplemental file.

Table of content:

P2: Supplemental figure 1

P3: Supplemental figure 2

P4: Supplemental figure 3

P5: Supplemental figure 4

P6: Supplemental figure 5

P7: Supplemental figure 6

P8: Supplemental table 1

P9: Supplemental table 2

P10-p14: Supplemental table 3

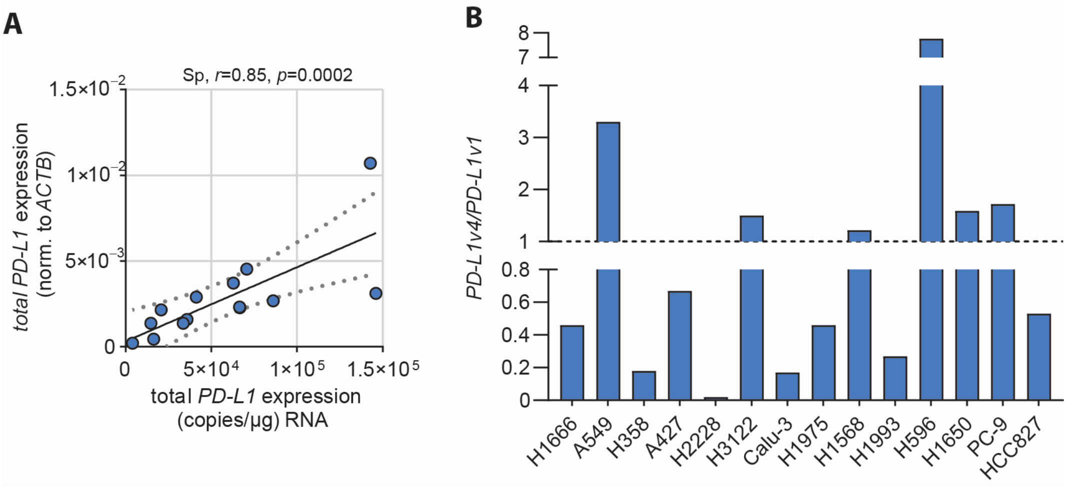

Supplemental Figure 1 (A) Correlation analyses of the total *PD-L1* expression determined in 14 NSCLC cell lines by RT-qPCR and by ddPCR. The Spearman correlation coefficient r is given. For the RT-qPCR experiments, *PD-L1* expression was normalized to the expression of *ACTB*. For ddPCR experiments, total *PD-L1* expression was determined from the addition of *PD-L1v1* and *PD-L1v4* expression and illustrated as copies of pr µg RNA. Depicted on the graph is linear best fit (solid line) and 95% confidence interval (dotted lines). **(B)** The *PD-L1v4/PD-L1v1* ratio was determined in 14 NSCLC cell lines from ddPCR expression analysis. *PD-L1v1* and *PD-L1v4* expression was measured in technical triplicates for each cell line and triplicates were merged before data analysis. To account for background unspliced mRNA and eventual contaminating DNA by the ddPCR assay detecting *PD-L1v4*, a ddPCR assay for *PD-L1* intron3 was applied to all samples. The resulting background measured by *PD-L1* intron3 (as copies pr µg RNA) was subtracted from the *PD-L1v4* expression (copies pr µg RNA) before calculation of the *PD-L1v4/PD-L1v1* ratio. For comparison, a *PD-L1v4/PD-L1v1* ratio of 1 is depicted on the graph as a dotted line.

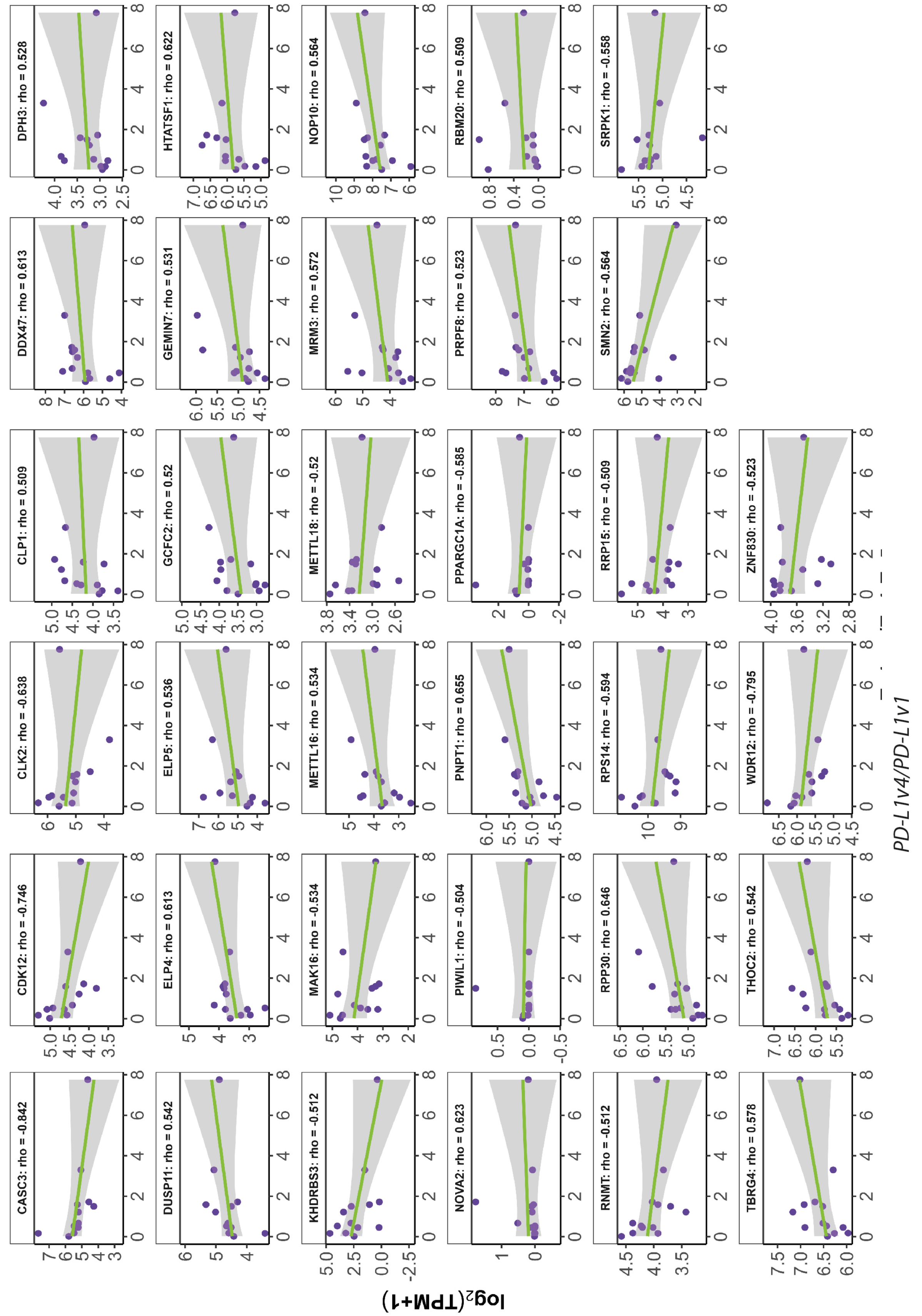

Supplemental Figure 2 Spearman correlation analysis between the expression of various factors extracted from DepMap Portal (TPM_22Q2) and the *PD-L1v4/PD-L1v1* ratio calculated from ddPCR analysis.

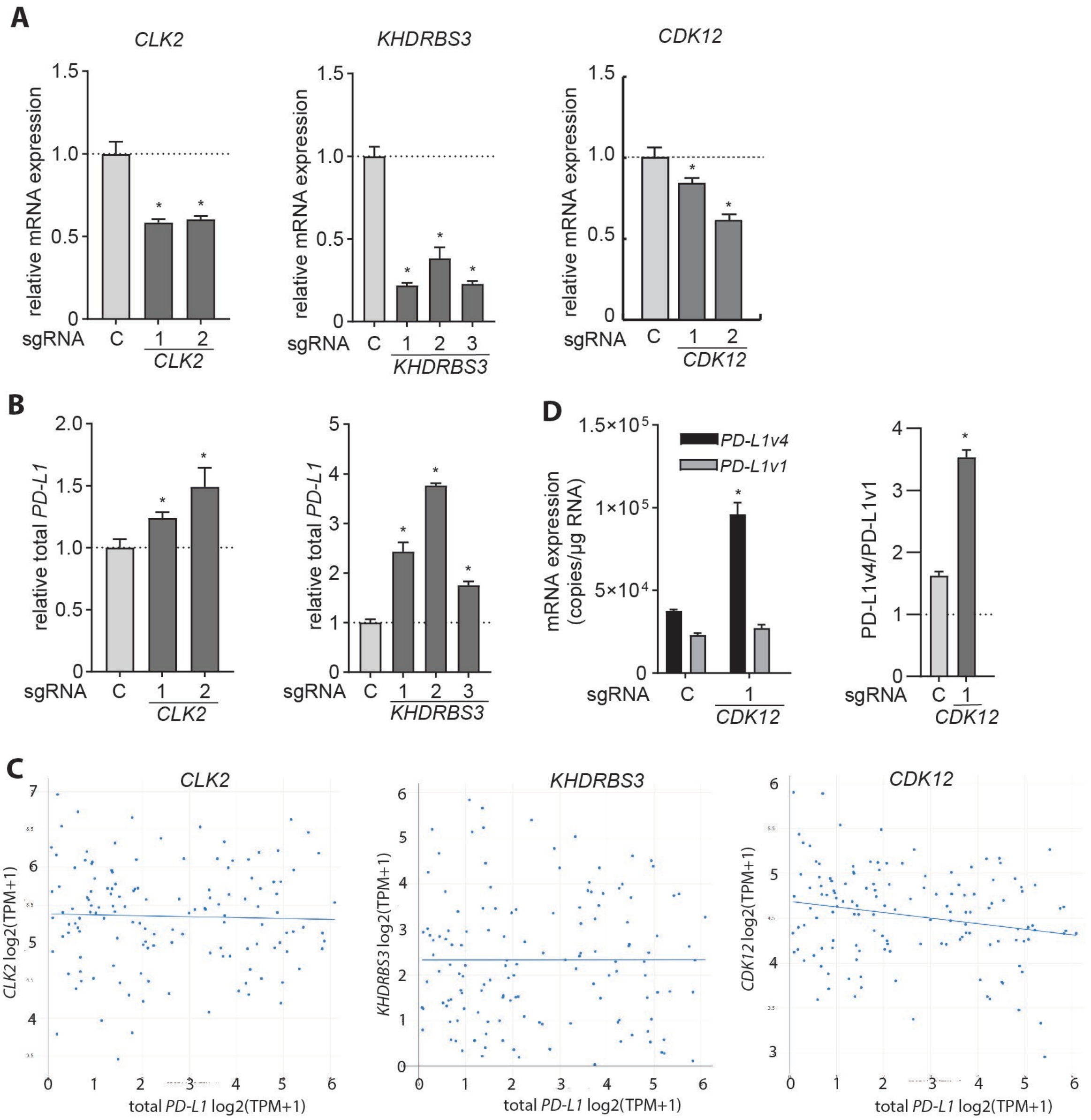

Supplemental Figure 3 (A) mRNA expression of *CLK2*, *KHDRBS3*, and *CDK12* in HCC827 cells following the introduction of indels with the given sgRNAs. Expression was calculated by RT-qPCR and normalized to *TBP* followed by normalization to control sgRNA (C) (as depicted by a dotted line). One-way ANOVA with Holm-Šídák multiple comparisons test was used to evaluate differences between C and gene-specific sgRNAs (n=3). *p<0.05. **(B)** The mRNA expression of total *PD-L1*, following the given CRISPR/Cas9-depletions in HCC827 cells. mRNA expression of total *PD-L1* was normalized to *TBP* followed by normalization to Control sgRNA (C) (as depicted by a dotted line). One-way ANOVA with Holm-Šídák multiple comparisons test was used to evaluate differences between C and gene-specific sgRNAs (n=3). *p<0.05. **(C)** Spearman correlation analysis between total *PD-L1* expression and the expression of the given factors in NSCLC cell lines. RNA-seq expression data were extracted from the Broad Dependency Map portal (n=137). **(D)** The effect of *CDK12* depletion in HCC827 cells for *PD-L1* expression and the *PD-L1v4*/*PD-L1v1* ratio with sgRNA 1 for *CDK12* (n=3). The expression levels were determined using ddPCR data. For each *PD-L1* mRNA variant, the effect of *CDK12* depletion was examined against C using two-way ANOVA with Holm-Šídák multiple comparisons test. The effect of *CDK12* depletion on the *PD-L1v4*/*PD-L1v1* ratio was examined using Students t-test. *p<0.05.

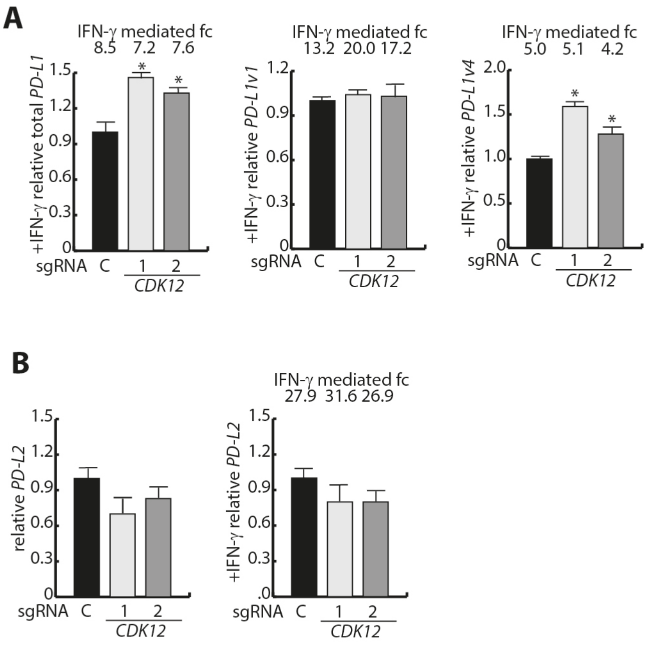

Supplemental Figure 4 (A) The mRNA expression of *PD-L1* variants was measured before and after IFN-γ stimulation for 24 h with RT-qPCR (n=2) in technical triplicates in HCC827 cells. HCC827 cells with the indicated sgRNA-mediated *CDK12* indels were analyzed. IFN-γ mediated fold change (fc) reports the effect of IFN-γ stimulation compared to untreated cells for each sgRNA (top panel). Fc for each sgRNA was subsequently normalized to sgRNA control. **(B)** Like panel (A) but with the examination of PD-L2 mRNA expression (n=1).

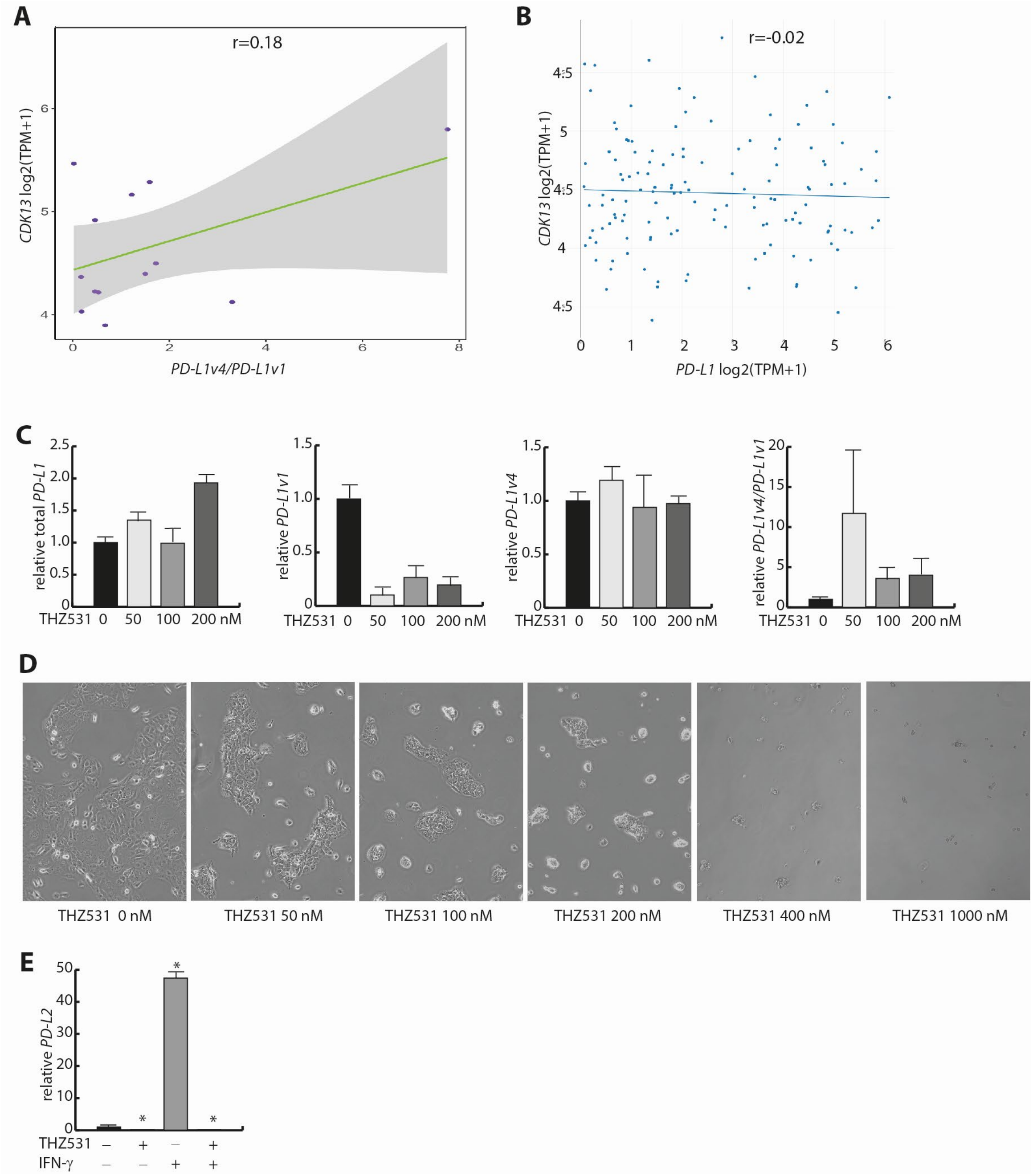

Supplemental Figure 5 (A) Spearman correlation analysis between the expression of *CDK13* and the *PD-L1v4/PD-L1v1* ratio. **(B)** Spearman correlation analysis between *CDK13* and total *PD-L1* from available expression data as presented in Supplemental Figure S3D. **(C)** The effect of supplementing HCC827 cells with various concentrations of THZ531 for 24 h for the expression of *PD-L1* mRNA variants and the *PD-L1v4/PD-L1v1* ratio. Expression was measured with RT-qPCR (n=1) in technical triplicates. **(D)** Cytotoxic effect of various THZ531 concentrations for 24 h in HCC827 cells. Cells were visualized with phase contrast microscopy using 5x objective. **(E)** The effect of supplementing HCC827 cells 100 nM THZ531 for 24 h and/or IFN-γ stimulation for 24 h for the expression of *PD-L2* mRNA. Expression was measured with RT-qPCR (n=1) in technical triplicates.

A

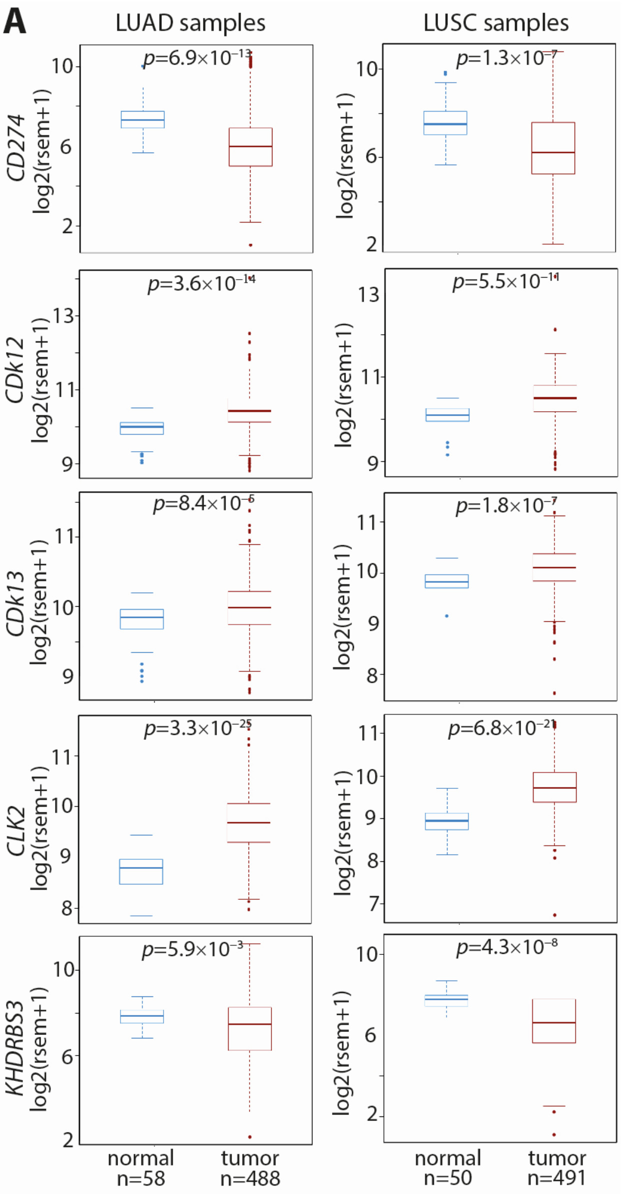

B

Spearman correlation r			*CDK12*	*CDK13*	*CLK2*	*KHDRBS3*
LUAD samples	normal n=58	*CD274*	0.48 *p*=6.3×10^{-5}	0.16 *p*=0.11	-0.19 *p*=0.07	-0.09 *p*=0.24
	tumor n=488	*CD274*	0.20 *p*=4.5×10^{-6}	0.07 *p*=0.06	-0.22 *p*=5.0×10^{-7}	-0.18 *p*=2.3×10^{-5}
LUSC samples	normal n=50	*CD274*	0.38 *p*=3.4×10^{-3}	-0.05 *p*=0.37	-0.36 *p*=5.4×10^{-3}	-0.08 *p*=0.29
	tumor n=491	*CD274*	0.11 *p*=7.7×10^{-3}	-0.00 *p*=0.45	-0.15 *p*=6.0×10^{-4}	-0.07 *p*=0.07

Supplemental Figure 6 (A) mRNA expression in TCGA LUAD and LUSC and corresponding normal lung tissue. mRNA expression levels are in format RNA-seq by expectation-maximization (RSEM) and Wilcoxon test adjusted *P*-values for differences between the groups. **(B)** Spearman correlation analyses for mRNA expression of total *PD-L1* and the given factors.

Supplemental Table S1 Primer sequences

	Gene	Site	Function	Sequence 5' – 3'	Tm
ddPCR	*PD-L1*	Exon3/Intron3	Forward	CGCTGCATGATCAGCTATGG	58
ddPCR	*PD-L1*	Exon3/Intron3	Reverse	TTTCAATAAAGATCAGGCCTCTCA	58
ddPCR	*PD-L1*	Exon3/Intron3	Probe	FAM-5'-CTTACCATTGACTTTCACAGTAATTCGCTTG-3'-BHQ1	58
ddPCR	*PD-L1*	Exon4/Exon5	Forward	GATCCTGAGGAAAACCATACAGC	60
ddPCR	*PD-L1*	Exon4/Exon5	Reverse	GGCTCCCAGAATTACCAAGTGA	60
ddPCR	*PD-L1*	Exon4/Exon5	Probe	HEX-5'-CATCCCAGAACTACCTCTGGCACATC-3'-BHQ1	60
ddPCR	*PD-L1*	Exon4A	Forward	ACAGCTGAATTGGTCATCCCAG	60
ddPCR	*PD-L1*	Exon4A	Reverse	AGGCAGACATCATGCTAGGTG	60
ddPCR	*PD-L1*	Exon4A	Probe	FAM-5'-TGTGTCCATTAAAATATGTCTAACACTGTCCCC-3'-BHQ1	60
ddPCR	*TBP*	Exon5/Exon6	Forward	AGGAGCCAAGAGTGAAGAACA	58
ddPCR	*TBP*	Exon6	Reverse	CCCAACTTCTGTACAACTCTAG	58
ddPCR	*TBP*	Exon6	Probe	HEX-5'-CATATTTTCTTGCTGCCAGTCTGGAC-3'-BHQ1	58
qPCR	*PD-L1*	Exon3	Forward	GGTGGTGCCGACTACAAGCGA	58
qPCR	*PD-L1*	Exon4	Reverse	TGACTTCGGCCTTGGGGTAGC	58
qPCR	*PD-L1*	Exon4/Exon5	Reverse	GGATGTGCCAGAGGTAGTTCTG	58
qPCR	*PD-L1*	Exon4A	Reverse	TTTTAATGGACACATTCAGAATATTACCTGG	58
qPCR	*PD-L1*	Exon3/Exon4	Forward	ACTGTGAAAGTCAATGCCCCA	58/60
qPCR	*PD-L1*	Exon4	Reverse	GGTGACTGGATCCACAACCA	58/60
qPCR	*ACTB*	Exon5	Forward	GGCGGCACCACCATGTACCCT	58/60
qPCR	*ACTB*	Exon6	Reverse	AGGGGCCGGACTCGTCATACT	58/60
qPCR	*TBP*	Exon5/Exon6	Forward	AGGAGCCAAGAGTGAAGAACAG	60
qPCR	*TBP*	Exon6	Reverse	CCCAACTTCTGTACAACTCTAGC	60
qPCR	*CDK12*	Exon6	Forward	CAGATTTTGGACTTGCTCGGC	60
qPCR	*CDK12*	Exon6/Exon7	Reverse	TCCCCAAGAATACATCCACAGC	60
qPCR	*CLK2*	Exon1	Forward	CGGACTTCCTGTGGGACAAG	60
qPCR	*CLK2*	Exon2	Reverse	GCTCCGATAGTGTTCACGGT	60
qPCR	*KHDRBS3*	Exon1	Forward	GAGGAGAAGTACCTGCCCGAG	58/60
qPCR	*KHDRBS3*	Exon2	Reverse	ACACTTTCTGTCCCAGCTTCAT	58/60
qPCR	*PD-L2*	Exon5	Forward	AGCCCTAAGAAAACAACTCTGTCA	60
qPCR	*PD-L2*	Exon6/Exon5	Reverse	ACAGGTCTTTTTGTTGTGT CTTTTG	60

Supplemental Table S2 sgRNA information

sgRNA information			ICE synthego analysis	
**Name**	**sgRNA oligonucleotides**	**Exon**	**Indel %**	**KO score %**
KHDRBS3 sgRNA ex1 SS	CACCGACCTTGGTTCACCAGGCGCA	Exon 1	96	61
KHDRBS3 sgRNA ex1 AS	AAACTGCGCCTGGTGAACCAAGGTC	Exon 1		
KHDRBS3 sgRNA ex3 SS	CACCGCGCTTCAGAGAATTGCCACG	Exon 3	90	78
KHDRBS3 sgRNA ex3 AS	AAACCGTGGCAATTCTCTGAAGCGC	Exon 3		
KHDRBS3 sgRNA ex5 SS	CACCGTACACCTCTTGTACGCAAGG	Exon 5	nd	
KHDRBS3 sgRNA ex5 AS	AAACCCTTGCGTACAAGAGGTGTAC	Exon 5		
CLK2 sgRNA ex2 SS	CACCGCTCACCTTCGAGAACGGACA	Exon 2	94	52
CLK2 sgRNA ex2 AS	AAACTGTCCGTTCTCGAAGGTGAGC	Exon 2		
CLK2 sgRNA ex3 SS	CACCGTGATCGTTCGTCCGACCGGA	Exon 3	70	66
CLK2 sgRNA ex3 AS	AAACTCCGGTCGGACGAACGATCAC	Exon 3		
CDK12 sgRNA 1 SS	CACCGACTTTGCAGCCGTCATCGGG	Exon 1	84	55
CDK12 sgRNA 1 AS	AAACCCCGATGACGGCTGCAAAGTC	Exon 1		
CDK12 sgRNA 2 SS	CACCGACCCCCGAAGCAGCATCCCT	Exon 1	73	51
CDK12 sgRNA 2 AS	AAACAGGGATGCTGCTTCGGGGGTC	Exon 1		

nd, not determined.

Supplemental Table S3 RNA processing family GO terms associated with the candidate genes correlating with the *PD-L1v4*/*PD-L1v1* mRNA ratio. For each gene the Spearman's rho, linear correlation coefficient, mean and SD of Log_2(TPM+1), GOIDs, and GO terms are reported.

Genes	Spearman's rho	Coefficient	Mean Log_2(TPM+1)	SD Log_2(TPM+1)	GOID	GO term
CASC3	-0.842	-0.172	5.328	0.817	GO:0000375	RNA splicing, via transesterification reactions
					GO:0000377	RNA splicing, via transesterification reactions with bulged adenosine as nucleophile
					GO:0000398	mRNA splicing, via spliceosome
					GO:0006396	RNA processing
					GO:0006397	mRNA processing
					GO:0008380	RNA splicing
CDK12	-0.746	-0.091	4.577	0.424	GO:0006396	RNA processing
					GO:0006397	mRNA processing
					GO:0008380	RNA splicing
					GO:0043484	regulation of RNA splicing
CLK2	-0.638	-0.074	5.257	0.656	GO:0006396	RNA processing
					GO:0008380	RNA splicing
					GO:0043484	regulation of RNA splicing
CLP1	0.509	0.023	4.199	0.472	GO:0000394	RNA splicing, via endonucleolytic cleavage and ligation
					GO:0006378	mRNA polyadenylation
					GO:0006388	tRNA splicing, via endonucleolytic cleavage and ligation
					GO:0006396	RNA processing
					GO:0006397	mRNA processing
					GO:0008033	tRNA processing
					GO:0008380	RNA splicing
					GO:0031123	RNA 3'-end processing
					GO:0031124	mRNA 3'-end processing
					GO:0034470	ncRNA processing
DDX47	0.613	0.087	6.054	0.873	GO:0006364	rRNA processing
					GO:0006396	RNA processing
					GO:0006397	mRNA processing
					GO:0008380	RNA splicing
					GO:0034470	ncRNA processing
DPH3	0.528	0.029	3.286	0.432	GO:0002097	tRNA wobble base modification
					GO:0002098	tRNA wobble uridine modification
					GO:0002926	tRNA wobble base 5-methoxycarbonylmethyl-2-thiouridinylation
					GO:0006396	RNA processing
					GO:0006400	tRNA modification
					GO:0008033	tRNA processing
					GO:0034470	ncRNA processing
DUSP11	0.542	0.087	4.603	0.462	GO:0006396	RNA processing
ELP4	0.613	0.109	3.568	0.491	GO:0002097	tRNA wobble base modification
					GO:0002098	tRNA wobble uridine modification
					GO:0006396	RNA processing
					GO:0006400	tRNA modification
					GO:0008033	tRNA processing
					GO:0034470	ncRNA processing
ELP5	0.536	0.141	5.166	0.868	GO:0002097	tRNA wobble base modification
					GO:0002098	tRNA wobble uridine modification
					GO:0006396	RNA processing
					GO:0006400	tRNA modification
					GO:0008033	tRNA processing
					GO:0034470	ncRNA processing
GCFC2	0.52	0.071	3.519	0.492	GO:0000245	spliceosomal complex assembly
					GO:0000375	RNA splicing, via transesterification reactions
					GO:0000377	RNA splicing, via transesterification reactions with bulged adenosine as nucleophile
					GO:0000398	mRNA splicing, via spliceosome
					GO:0006396	RNA processing
					GO:0006397	mRNA processing

					GO:0008380	RNA splicing
GEMIN7	0.531	0.06	4.991	0.455	GO:0000375	RNA splicing, via transesterification reactions
					GO:0000377	RNA splicing, via transesterification reactions with bulged adenosine as nucleophile
					GO:0000387	spliceosomal snRNP assembly
					GO:0000398	mRNA splicing, via spliceosome
					GO:0006396	RNA processing
					GO:0006397	mRNA processing
					GO:0008380	RNA splicing
HTATSF1	0.622	0.047	5.893	0.532	GO:0000245	spliceosomal complex assembly
					GO:0000375	RNA splicing, via transesterification reactions
					GO:0000377	RNA splicing, via transesterification reactions with bulged adenosine as nucleophile
					GO:0000398	mRNA splicing, via spliceosome
					GO:0006396	RNA processing
					GO:0006397	mRNA processing
					GO:0008380	RNA splicing
					GO:1903241	U2-type prespliceosome assembly
KHDRBS3	-0.512	-0.36	2.214	1.462	GO:0000375	RNA splicing, via transesterification reactions
					GO:0000377	RNA splicing, via transesterification reactions with bulged adenosine as nucleophile
					GO:0000380	alternative mRNA splicing, via spliceosome
					GO:0000381	regulation of alternative mRNA splicing, via spliceosome
					GO:0000398	mRNA splicing, via spliceosome
					GO:0006396	RNA processing
					GO:0006397	mRNA processing
					GO:0008380	RNA splicing
					GO:0043484	regulation of RNA splicing
					GO:0048024	regulation of mRNA splicing, via spliceosome
					GO:0050684	regulation of mRNA processing
MAK16	-0.534	-0.113	3.977	0.697	GO:0000460	maturation of 5.8S rRNA
					GO:0000470	maturation of LSU-rRNA
					GO:0006364	rRNA processing
					GO:0006396	RNA processing
					GO:0034470	ncRNA processing
METTL16	0.534	0.101	3.815	0.668	GO:0000154	rRNA modification
					GO:0000375	RNA splicing, via transesterification reactions
					GO:0000377	RNA splicing, via transesterification reactions with bulged adenosine as nucleophile
					GO:0000398	mRNA splicing, via spliceosome
					GO:0006364	rRNA processing
					GO:0006396	RNA processing
					GO:0006397	mRNA processing
					GO:0008380	RNA splicing
					GO:0031167	rRNA methylation
					GO:0034470	ncRNA processing
					GO:0043484	regulation of RNA splicing
					GO:0048024	regulation of mRNA splicing, via spliceosome
					GO:0050684	regulation of mRNA processing
					GO:0070475	rRNA base methylation
METTL18	-0.52	-0.026	3.189	0.34	GO:0006364	rRNA processing
					GO:0006396	RNA processing
					GO:0034470	ncRNA processing
					GO:2000232	regulation of rRNA processing
MRM3	0.572	0.092	4.225	0.698	GO:0000154	rRNA modification
					GO:0000451	rRNA 2'-O-methylation
					GO:0006364	rRNA processing
					GO:0006396	RNA processing
					GO:0031167	rRNA methylation

					GO:0034470	ncRNA processing
NOP10	0.564	0.16	7.827	0.789	GO:0000154	rRNA modification
					GO:0006364	rRNA processing
					GO:0006396	RNA processing
					GO:0031118	rRNA pseudouridine synthesis
					GO:0034470	ncRNA processing
NOVA2	0.623	0.023	0.224	0.486	GO:0000375	RNA splicing, via transesterification reactions
					GO:0000377	RNA splicing, via transesterification reactions with bulged adenosine as nucleophile
					GO:0000380	alternative mRNA splicing, via spliceosome
					GO:0000381	regulation of alternative mRNA splicing, via spliceosome
					GO:0000398	mRNA splicing, via spliceosome
					GO:0006396	RNA processing
					GO:0006397	mRNA processing
					GO:0008380	RNA splicing
					GO:0043484	regulation of RNA splicing
					GO:0048024	regulation of mRNA splicing, via spliceosome
					GO:0050684	regulation of mRNA processing
PIWIL1	-0.504	-0.005	0.08	0.233	GO:0006396	RNA processing
					GO:0034470	ncRNA processing
					GO:0034587	piRNA processing
					GO:0070918	regulatory ncRNA processing
PNPT1	0.655	0.083	5.147	0.321	GO:0000963	mitochondrial RNA processing
					GO:0000964	mitochondrial RNA 5'-end processing
					GO:0000965	mitochondrial RNA 3'-end processing
					GO:0000966	RNA 5'-end processing
					GO:0006378	mRNA polyadenylation
					GO:0006396	RNA processing
					GO:0006397	mRNA processing
					GO:0031123	RNA 3'-end processing
					GO:0031124	mRNA 3'-end processing
					GO:0090615	mitochondrial mRNA processing
					GO:0090616	mitochondrial mRNA 3'-end processing
					GO:0097222	mitochondrial mRNA polyadenylation
PPARGC1A	-0.585	-0.065	0.535	0.955	GO:0006396	RNA processing
					GO:0006397	mRNA processing
					GO:0008380	RNA splicing
PRPF8	0.523	0.095	6.942	0.595	GO:0000244	spliceosomal tri-snRNP complex assembly
					GO:0000375	RNA splicing, via transesterification reactions
					GO:0000377	RNA splicing, via transesterification reactions with bulged adenosine as nucleophile
					GO:0000387	spliceosomal snRNP assembly
					GO:0000398	mRNA splicing, via spliceosome
					GO:0006396	RNA processing
					GO:0006397	mRNA processing
					GO:0008380	RNA splicing
RBM20	0.509	0.017	0.255	0.316	GO:0000375	RNA splicing, via transesterification reactions
					GO:0000377	RNA splicing, via transesterification reactions with bulged adenosine as nucleophile
					GO:0000380	alternative mRNA splicing, via spliceosome
					GO:0000381	regulation of alternative mRNA splicing, via spliceosome
					GO:0000398	mRNA splicing, via spliceosome
					GO:0006396	RNA processing
					GO:0006397	mRNA processing
					GO:0008380	RNA splicing
					GO:0033119	negative regulation of RNA splicing
					GO:0033120	positive regulation of RNA splicing
					GO:0043484	regulation of RNA splicing

					GO:0048024	regulation of mRNA splicing, via spliceosome
					GO:0048025	negative regulation of mRNA splicing, via spliceosome
					GO:0050684	regulation of mRNA processing
					GO:0050686	negative regulation of mRNA processing
RNMT	-0.512	-0.048	4.043	0.31	GO:0006370	7-methylguanosine mRNA capping
					GO:0006396	RNA processing
					GO:0006397	mRNA processing
					GO:0009452	7-methylguanosine RNA capping
					GO:0036260	RNA capping
					GO:0106005	RNA 5'-cap (guanine-N7)-methylation
RPP30	0.646	0.08	5.214	0.4	GO:0000966	RNA 5'-end processing
					GO:0001682	tRNA 5'-leader removal
					GO:0006364	rRNA processing
					GO:0006396	RNA processing
					GO:0008033	tRNA processing
					GO:0034470	ncRNA processing
					GO:0099116	tRNA 5'-end processing
RPS14	-0.594	-0.068	9.772	0.506	GO:0000462	maturation of SSU-rRNA from tricistronic rRNA transcript (SSU-rRNA, 5.8S rRNA, LSU-rRNA)
					GO:0006364	rRNA processing
					GO:0006396	RNA processing
					GO:0030490	maturation of SSU-rRNA
					GO:0034470	ncRNA processing
RRP15	-0.509	-0.074	4.241	0.657	GO:0000460	maturation of 5.8S rRNA
					GO:0000470	maturation of LSU-rRNA
					GO:0006364	rRNA processing
					GO:0006396	RNA processing
					GO:0034470	ncRNA processing
SMN2	-0.564	-0.292	5.053	0.995	GO:0000245	spliceosomal complex assembly
					GO:0000375	RNA splicing, via transesterification reactions
					GO:0000377	RNA splicing, via transesterification reactions with bulged adenosine as nucleophile
					GO:0000387	spliceosomal snRNP assembly
					GO:0000398	mRNA splicing, via spliceosome
					GO:0006396	RNA processing
					GO:0006397	mRNA processing
					GO:0008380	RNA splicing
SRPK1	-0.558	-0.042	5.232	0.379	GO:0000245	spliceosomal complex assembly
				0.379	GO:0000375	RNA splicing, via transesterification reactions
				0.379	GO:0000377	RNA splicing, via transesterification reactions with bulged adenosine as nucleophile
				0.379	GO:0000398	mRNA splicing, via spliceosome
				0.379	GO:0006396	RNA processing
				0.379	GO:0006397	mRNA processing
				0.379	GO:0008380	RNA splicing
				0.379	GO:0043484	regulation of RNA splicing
				0.379	GO:0048024	regulation of mRNA splicing, via spliceosome
				0.379	GO:0050684	regulation of mRNA processing
TBRG4	0.578	0.076	6.552	0.368	GO:0000963	mitochondrial RNA processing
					GO:0006396	RNA processing
					GO:0006397	mRNA processing
					GO:0090615	mitochondrial mRNA processing
THOC2	0.542	0.088	5.846	0.411	GO:0006396	RNA processing
					GO:0006397	mRNA processing
					GO:0008380	RNA splicing
WDR12	-0.795	-0.06	5.809	0.418	GO:0000460	maturation of 5.8S rRNA
					GO:0000463	maturation of LSU-rRNA from tricistronic rRNA transcript (SSU-rRNA, 5.8S rRNA, LSU-rRNA)
					GO:0000466	maturation of 5.8S rRNA from tricistronic rRNA transcript (SSU-rRNA, 5.8S rRNA, LSU-rRNA)

					GO:0000470	maturation of LSU-rRNA
					GO:0006364	rRNA processing
					GO:0006396	RNA processing
					GO:0034470	ncRNA processing
ZNF830	-0.523	-0.035	3.653	0.305	GO:0006396	RNA processing
					GO:0006397	mRNA processing
					GO:0008380	RNA splicing
